# The Relationship between Trait Emotional Intelligence and Creative Self-Efficacy in Gifted Children: A Cross-Lagged and Cross-Temporal Mediation Analysis

**DOI:** 10.3390/jintelligence12080071

**Published:** 2024-07-23

**Authors:** Li Cheng, Xinmei Liu, Yujuan Liu, Yilin Wu

**Affiliations:** 1 Faculty of Education, Beijing Normal University, Beijing 100875, China; xmliu@mail.bnu.edu.cn; 2 Developmental and Educational Research Center of Children’s Creativity, Faculty of Education, Beijing Normal University, Beijing 100875, China; 3 Institute of Psychology and Special Education, China National Academy of Educational Sciences, Beijing 100088, China; liuyj@cnaes.edu.cn

**Keywords:** gifted children, creative self-efficacy, trait emotional intelligence, self-concept

## Abstract

The present study aimed to investigate the causal relationship between trait emotional intelligence and creative self-efficacy in gifted children and to explore the cross-temporal mediating role of self-concept between these two variables. A total of 177 gifted children aged 10–13 years (*M* = 11.29, *SD* = 0.68) were selected from an experimental class of gifted children in a middle school. The results showed that (1) the trait emotional intelligence and creative self-efficacy of gifted children decreased with age and that (2) trait emotional intelligence at time 1 (T1) positively and significantly predicted creative self-efficacy at time 2 (T2). The Self-Description Questionnaire was added at the second follow-up, which revealed that (3) T2 non-academic self-concept fully mediated the relationship between T1 trait emotional intelligence and T2 creative self-efficacy. This study revealed a lasting positive effect of trait emotional intelligence on the development of creative self-efficacy in gifted children. Additionally, trait emotional intelligence was found to indirectly influence creative self-efficacy through non-academic self-concept.

## 1. Introduction

Gifted children, who exhibit exceptional abilities or potential in areas such as intelligence and creativity, represent a vital reserve of innovative talents and are essential human resources for driving social development (Cheng et al. 2023). Creativity, as one of the significant characteristics of gifted children (Renzulli 2011), is defined as the ability of an individual to utilize existing environmental resources and personal experience to generate a novel, unique, appropriate, and valuable product (Runco and Jaeger 2012; Zha et al. 2006). A crucial aspect of enhancing creativity lies in encouraging and fortifying an individual’s creative self-efficacy (Karwowski and Lebuda 2013). Creative self-efficacy pertains to an individual’s confidence and belief in his or her capacity to generate innovative and adaptable ideas, problem-solving strategies, and behavioral performance (Beghetto 2009; Zhang et al. 2021). Researchers have increasingly focused their attention on examining the correlation between an individual’s emotional state and his or her creative self-efficacy (Karwowski et al. 2019). Trait emotional intelligence refers to an individual’s self-perception and behavioral tendencies in recognizing, processing, and utilizing emotional information. Social and emotional skills are widely acknowledged as crucial for both academic achievement and personal growth in the 21st century. Studies have demonstrated that emotional experiences can have a lasting impact on an individual’s overall functioning (Culot and Gevers 2021; He and Wong 2022). Social cognitive theory provides valuable insights into the intersection of trait emotional intelligence and creative self-efficacy (Bandura 1986), suggesting that positive emotional experiences facilitate the development of creative self-efficacy, while negative emotional experiences hinder it (He and Wong 2022). Gifted children often display traits such as hypersensitivity and hyperexcitability (Liu and Chen 2021; Mendaglio 1995), which can impact their emotional well-being during tasks (Cornoldi et al. 2021; Zeidner and Matthews 2017). This, in turn, can affect their creative achievements in comparison to those of their peers. Furthermore, their creative self-efficacy, a crucial component of their overall self-confidence, is closely linked to their general self-concept. Trait emotional intelligence is a crucial psychological aspect of human development that plays a pivotal role in shaping one’s self-concept (Martínez-Monteagudo et al. 2021).

Notably, indicators such as creative self-efficacy, trait emotional intelligence, and self-concept have frequently been employed in various studies to assess the mental health and psychological adjustment of gifted children (Cheng et al. 2022; Neihart 1999). Due to their unique social experiences and emotional characteristics, psychosocial skills play a crucial role in shaping their mental health. This study will further explore the relationship between trait emotional intelligence, creative self-efficacy, and self-concept in gifted children, to provide insights into the balanced development of gifted children.

### 1.1. Trait Emotional Intelligence and Creative Self-Efficacy

Social intelligence, as defined in Thorndike and Stein’s (1937) multifactorial theory of intelligence, and interpersonal intelligence and self-knowledge, as defined in Gardner’s (1983) multiple theories of intelligence, are considered vital elements in shaping the connotations of emotional intelligence (Peng et al. 2004). The mixed model of emotional intelligence proposed by Bar-On (2004, 2006) categorizes emotional intelligence into five dimensions, which are the intra-individual component, the interpersonal component, the stress-management component, the adaptive component, and the general state of mind. The model emphasizes that emotional intelligence is a combination of cognitive and non-cognitive factors. Based on the mixed model of emotional intelligence, emotional intelligence is a composite structure that includes personality traits, emotions, motivation, and self-perception abilities (Bar-On 2004), and it affects an individual’s ability to successfully cope with environmental demands and stress challenges. This conceptual framework is known as trait emotional intelligence, highlighting the link between emotional intelligence and personality traits (Bar-On 2006; Chen and Cheng 2023). Trait emotional intelligence can help individuals enhance the effectiveness of problem solving.

Existing studies have focused on gifted children in regular classes to explore the developmental trends of trait emotional intelligence and creative self-efficacy. For instance, scholars have indicated that emotional perception and understanding, as well as reasoning of their own emotions, show a linear increase for gifted children in regular classes over a one-year tracking period (Chen 2024). Another study investigating creative self-efficacy has revealed that the probability of gifted children in regular classes maintaining a high level of creative self-efficacy throughout their development is 60%, with a 37% chance of transitioning from a low level to a high level (Geng 2023). In addition, drawing from social comparison theory, Marsh and Parker (1984) proposed the big-fish–little-pond effect (BFLPE), which suggests that students with the same level of ability tend to exhibit a lower academic self-concept when placed in high-ability classes or schools. They are more likely to perceive themselves as not outstanding. In contrast, students in schools or classes with lower standards have a higher academic self-concept, feeling more confident and optimistic. Based on the big-fish–little-pond effect (BFLPE), the gifted children in this study, who are concentrated in an experimental class for gifted children, may exhibit a declining trend of trait emotional intelligence and creative self-efficacy (Li and Shi 2005). Upon entering the experimental class, gifted children may face academic challenges when comparing themselves to their equally intellectually gifted peers. This comparison may result in a loss of confidence in their academic abilities and lower levels of self-evaluation (Chen and Shi 2013; Duan et al. 2022), and it can also have a negative impact on the development of trait emotional intelligence and creative self-efficacy in gifted children. Accordingly, we proposed Hypothesis 1: The levels of emotional intelligence and creative self-efficacy of gifted children exhibit a declining pattern across two time points.

Additionally, there is a close correlation between trait emotional intelligence and creative thinking and creative ability (Meilstrup et al. 2020; Supervía and Robres 2021; Wang et al. 2020; Wu et al. 2019). However, scholars have different opinions about the cause-and-effect relationship between the two, and these perspectives are summarized in two main views. On the one hand, trait emotional intelligence can enhance the development of self-efficacy, and this conclusion has been verified across diverse groups. Supervía and Robres (2021) conducted a study involving 2204 secondary school students and found that emotion regulation significantly predicted self-efficacy. In another study, Wu et al. (2019) investigated 497 secondary school teachers and revealed that teachers’ trait emotional intelligence can effectively promote their self-efficacy. On the other hand, the contribution of self-efficacy to the development of trait emotional intelligence has also been demonstrated in multiple groups. For example, Meilstrup et al. (2020) conducted a study with 3969 elementary and secondary school students in grades 5–9 and discovered that high self-efficacy mediated the negative effects of low socioeconomic status on the emotional states of school-age children. Wang et al. (2020) randomly selected 835 college students from two comprehensive universities and found that general self-efficacy significantly predicted trait emotional intelligence. The comprehensive findings from previous research suggest that there may be a reciprocal predictive relationship between trait emotional intelligence and creative self-efficacy, providing a basis for investigating the connection between trait emotional intelligence and creative self-efficacy in gifted children. Therefore, in this study, we proposed Hypothesis 2: There is a mutual predictive effect between trait emotional intelligence and creative self-efficacy in gifted children.

### 1.2. Self-Concept as the Link between Trait Emotional Intelligence and Creative Self-Efficacy

The multidimensional and hierarchical model of self-concept proposed by Shavelson et al. (1976) categorizes general self-concept into academic self-concept and non-academic self-concept. As a core component of personality, self-concept refers to individuals’ understanding and perception of various aspects of themselves that gradually form through interactions with both the subjective and objective world as well as interpersonal relationships (Byrne 1986). Individuals with a positive self-concept are capable of making objective evaluations of themselves and accepting themselves in a positive way (Chen et al. 2010). Existing research indicates that there is a close relationship between an individual’s self-concept and their trait emotional intelligence and creative self-efficacy (Bandura 1986; Chen and Cheng 2023; Fernández-Berrocal and Extremera 2006; Martínez-Monteagudo et al. 2021).

According to social cognitive theory, trait emotional intelligence, as an important psychological construct for individual development, is closely related to an individual’s self-concept (Bandura 1986; Chen and Cheng 2023). Trait emotional intelligence pertains to an individual’s ability to accurately interpret emotional information in social contexts, encompassing self-awareness and tendencies related to emotions (Fiorilli et al. 2020). Individuals with a higher level of trait emotional intelligence exhibit superior skill in emotion management and regulation, which consequently fosters greater self-awareness (Fernández-Berrocal and Extremera 2006). This self-awareness effectively promotes the positive development of one’s self-concept. Studies have shown that if individuals are able to process negative emotions and maintain a positive mindset in unfavorable situations, they can sustain a high level of self-concept (Martínez-Monteagudo et al. 2021). Conde-Pipó et al. (2021) conducted a study involving 520 adults aged between 41 and 80 years and found that factors such as intrinsic motivation and emotion regulation can contribute to maintaining a positive level of body self-concept (non-academic self-concept).

Creative self-efficacy is derived from general self-efficacy, and an individual’s level of self-concept significantly influences the development of their creative self-efficacy. Scholars have highlighted the pivotal role of self-concept in shaping self-efficacy (Arens et al. 2022). Corresponding research findings also indicate that there is a significant positive correlation between self-concept and self-efficacy, and students’ academic self-concept strongly influences their academic self-efficacy beliefs. Moreover, an individual’s non-academic self-concept also plays a positive role in promoting their creative self-efficacy. A warm and supportive parent–child relationship provides a secure foundation for an individual to develop; when individuals feel supported and encouraged from their parents, they are more likely to believe that they can succeed in creative tasks (Halford et al. 2018). Furthermore, when individuals are satisfied with their physical appearance, they are more likely to feel confident and valuable, which translates into confidence in their ability to perform creative activities (Simon et al. 2022).

The above findings suggest that both academic self-concept and non-academic self-concept may serve as mediating factors between trait emotional intelligence and creative self-efficacy. Moreover, scholars have conducted a one-year longitudinal study on gifted children in experimental and regular classes. The findings revealed that the academic self-concept of gifted children in the experimental class declined after 6 and 12 months, while non-academic self-concept did not change significantly (Chessor and Whitton 2005). In addition, the BFLPE is primarily observed in academic self-concept, with evidence suggesting that academic self-concept decreased in the gifted children who enter the experimental classes (Luo et al. 2008). The different developmental trends of gifted children in academic and non-academic self-concepts may lead to the possibility that academic and non-academic self-concepts play different mediating roles between trait emotional intelligence and creative self-efficacy, which deserves to be further explored. Therefore, we propose research Hypothesis 3: Academic self-concept and non-academic self-concept play different mediating roles between trait emotional intelligence and creative self-efficacy in gifted children.

In summary, existing research has explored the developmental trends of trait emotional intelligence and creative self-efficacy among gifted children in regular classes, and the results of the relationship between the two are inconsistent. Moreover, gifted children have different levels in academic self-concept and non-academic self-concept, and the role of academic self-concept and non-academic self-concept in the relationship between trait emotional intelligence and creative self-efficacy needs to be further explored. Therefore, this study will collect data at two different time points, with a measurement interval of 9 months, to explore the developmental trends of trait emotional intelligence and creative self-efficacy among gifted children in an experimental class. It will further analyze the relationship between trait emotional intelligence and creative self-efficacy. Based on the analysis, we will analysis the cross-temporal mediating effects of academic and non-academic self-concepts on trait emotional intelligence and creative self-efficacy, respectively. Resolving these issues will not only provide further validation of the social cognitive theory and the BFLPE from the perspective of the gifted children in the experimental class but will offer references and insights for the scientific placement of gifted children and the promotion of their healthy development of non-cognitive abilities.

## 2. Materials and Methods

### 2.1. Participants

The participants in this study were recruited from an experimental class of gifted children’s education in a secondary school in North China. Based on The Three Ring Conception of Giftedness, it is recommended that above-average ability, creativity, and task commitment are important criteria for identifying gifted children. According to this model, the selection of gifted children in this school was carried out systematically and scientifically based on their physical and mental development characteristics. The selection process consisted of three rounds of procedures, comprising preliminary testing, retesting, and dynamic evaluation, to comprehensively and objectively assess children’s cognitive ability, developmental potential, academic level, and comprehensive qualities. A total of 177 gifted children (children with IQ test scores in the top 10% of the Beijing normative sample) aged 10–13 years, with a mean age of 11.29 years (*SD* = 0.68), were selected, including 76 girls (42.94%).

### 2.2. Materials

#### 2.2.1. Emotional Intelligence Scale (EIS)

The Emotional Intelligence Scale (EIS), developed by Schutte et al. (1998), is a self-reporting scale that is one of the most widely used scales in the field of trait emotional intelligence research. The Chinese version of the scale was revised by Wang and He (2002). We used the Chinese version to assess the trait emotional intelligence of the participants. The Chinese version consists of 33 questions divided into four dimensions: emotion perception (e.g., “I am aware of my emotions as I experience them”), managing self-relevant emotions (e.g., “I seek out activities that make me happy”), managing others’ emotions (e.g., “I arrange events others enjoy”), and emotion utilization (e.g., “When I feel a change in emotions, I tend to come up with new ideas”). Participants were asked to rate the items on a 5-point scale ranging from 1 (strongly disagree) to 5 (strongly agree), with questions 5, 28, and 33 being reverse-scored questions. Higher scores indicate higher levels of trait emotional intelligence. In this study, the Cronbach’s alpha coefficients of the two measurements were 0.93 and 0.94, respectively.

#### 2.2.2. Creative Self-Efficacy Scale (CSES)

The Creative Self-Efficacy Scale (CSES), developed by Tierney and Farmer (2002), was used to assess creative self-efficacy in gifted children. We used the Chinese version (Chen and Cheng 2023) to measure the creative self-efficacy of the participants. The scale consists of three items (e.g., “I have a lot of good ideas”). Participants were asked to rate these items on a 5-point scale ranging from 1 (strongly disagree) to 5 (strongly agree). Higher scores indicate higher levels of individual creative self-efficacy. In this study, the Cronbach’s alpha coefficients of the two measurements were 0.82 and 0.87, respectively.

#### 2.2.3. Self-Description Questionnaire (SDQ-II)

The Self-Description Questionnaire (SDQ-II), developed by Marsh et al. (1988), is commonly used to measure students’ level of self-concept development. We used the Chinese version (Dong et al. 1993) to measure the self-descriptions of the participants. The scale consists of 62 questions and includes two parts: academic self-concept, which includes three dimensions—reading proficiency (e.g., “I’m good at reading”), mathematics proficiency (e.g., “I’m looking forward to math class”), and general school performance (e.g., “I’m interested in all the courses”)—and non-academic self-concept, which includes four dimensions—peer relationships (e.g., “I have a lot of friends”), parent–child relationships (e.g., “My parents understand me”), physical appearance (e.g., “I’m good-looking”), and sports (e.g., “I’m good at sports”). Participants were asked to rate the items on a 5-point scale ranging from 1 (strongly disagree) to 5 (strongly agree), with questions 6, 18, 20, 28, 41, 56, and 62 being reverse-scored questions. Higher scores indicate higher levels of self-description. In this study, the Cronbach’s alpha coefficient was 0.93 for the academic self-concept subscale and 0.94 for the non-academic self-concept subscale.

### 2.3. Procedures

The study was approved by the Research Ethics Committee of the Department of Education, Beijing Normal University (protocol code: BNU202106100016). A team of postgraduate students from Beijing Normal University who received training conducted the measurements. Before collecting the data, informed consent was obtained from both the school officials and the students themselves. The data were collected twice; the first test was in September 2021 when the participants had just entered the gifted children’s experimental class, and the second test was conducted in early June 2022, with a 9-month gap between the tests, using a group-administered format. The same testing procedure was followed on both occasions, with the addition of the SDQ-II during the second test. Prior to taking the test, the students received detailed instructions on how to complete the questionnaire. They were assured that the results would only be used for research purposes and that their responses would remain confidential, and they were encouraged to answer the questionnaire honestly. The students were given 30 min to complete the questionnaire, which they returned immediately.

### 2.4. Data Analysis

In this study, we used SPSS 26.0 and AMOS 24.0 to analyze the data. First, SPSS 26.0 was used to perform Harman’s one-way test, descriptive analysis, and Pearson’s correlation analysis, and a repeated-measures analysis of variance (ANOVA) on the data. Second, based on the results of the correlation analysis, a cross-lagged model was constructed in AMOS 24.0. When performing the structural equation modeling, the maximum likelihood was used for fitting the SEM models; the model fit was considered acceptable if the comparative fit index (CFI) ≥ 0.90 and the root mean square error of approximation (RMSEA) < 0.08 and deemed good if CFI ≥ 0.95 and RMSEA ≤ 0.06 (Brown 2015; Watkins and Styck 2017).

## 3. Results

### 3.1. Common Method Bias Test

This study employed Harman’s single-factor analysis to test for the presence of common method bias. All variables were subjected to exploratory factor analysis to examine the unrotated factor analysis results. At T1, 8 factors with eigenvalues greater than 1 were extracted, and the maximum factor variance explained was 31.61% (less than 40%). At T2, 21 factors with eigenvalues greater than 1 were extracted, and the maximum factor variance explained was 27.46% (less than 40%). Therefore, this study does not exhibit severe common method bias (Podsakoff et al. 2003).

### 3.2. Descriptive Analysis and Correlation Analysis

Table 1 shows the mean (M), standard deviation (SD), and correlations of the variables for the two measurements. Creative self-efficacy showed significant positive correlations with trait emotional intelligence at both the T1 and T2 time points, and there is a significant positive correlation between T1 emotional intelligence and T2 self-concept, as well as between T2 self-concept and T2 creative self-efficacy.

A repeated-measures analysis of variance (ANOVA) was conducted with measurement time 1 and time 2 as the within-subjects variable, and trait emotional intelligence and creative self-efficacy as the dependent variables. The results showed that the main effect of measurement time was statistically significant. There was a statistically significant decrease in trait emotional intelligence from time 1 (*M* = 3.86, *SD* = 0.57) to time 2 (*M* = 3.76, *SD* = 0.58) (*F*_(1,174)_ = 8.34, *p* = .004, ηp^2^ = 0.04), and the results also showed there was a statistically significant decrease in creative self-efficacy from time 1 (*M* = 3.88, *SD* = 0.80) to time 2 (*M* = 3.59, *SD* = 0.94) (*F*_(1,174)_ = 17.31, *p* < .001, ηp^2^ = 0.09). After the gifted children entered the experimental class, their trait emotional intelligence and creative self-efficacy decreased over time.

### 3.3. Cross-Lagged Analysis of Trait Emotional Intelligence and Creative Self-Efficacy in Gifted Children

**Figure 1:**
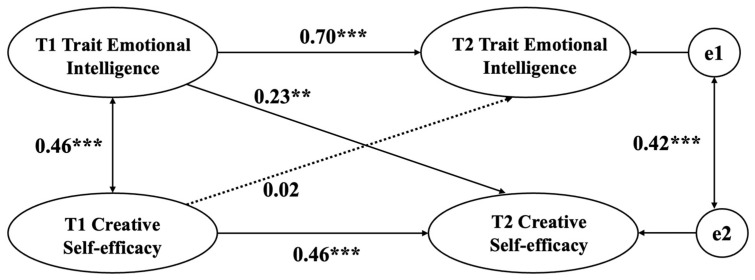
Cross-lagged model of trait emotional intelligence and creative self-efficacy. ** *p* < .01, *** *p* < .001. T1 = time 1, T2 = time 2.

We examined the reciprocal predictive role between trait emotional intelligence and creative self-efficacy in gifted children by constructing a cross-lagged model in AMOS 24.0 (as shown in Figure 1).The model fit was acceptable, with CFI = 0.96, and RMSEA = 0.07 (90% confidence interval (CI), 0.052–0.090).

The autoregressions of trait emotional intelligence and creative self-efficacy were significant between T1 and T2, suggesting some degree of stability across time for trait emotional intelligence (β = 0.70, *p* < .001) and creative self-efficacy (β = 0.46, *p* < .001) as gifted students aged. Cross-lagged analyses revealed that T1 trait emotional intelligence significantly and positively predicted T2 creative self-efficacy (β = 0.23, *p* = .006), but T1 creative self-efficacy did not significantly predict T2 trait emotional intelligence (β = 0.02, *p* = .81). This result indicated that there is a unidirectional predictive relationship between trait emotional intelligence and creative self-efficacy in gifted children, i.e., trait emotional intelligence positively and significantly predicts creative self-efficacy after 9 months.

### 3.4. Cross-Temporal Mediating Role of Self-Concept in Gifted Children

According to social cognitive theory, there is a close relationship between self-concept, trait emotional intelligence, and creative self-efficacy in gifted children. Therefore, building upon the findings of the cross-lagged analyses described above, we further explored the cross-temporal mediating effect of T2 self-concept. The mediation model was constructed in AMOS 24.0, aiming to test the mediating effect of T2 self-concept (academic self-concept and non-academic self-concept) between T1 trait emotional intelligence and T2 creative self-efficacy by using the bias-corrected percentile bootstrap method. Based on the method published by Bollen and Stine (1990), 5000 resampling iterations of the original data (N = 177) were used to calculate the 95% confidence interval to test the mediating effect of the model; if the confidence interval did not include 0, it indicated the existence of a mediating effect. The model fit was acceptable, with CFI = 0.96, and RMSEA = 0.07 (90% confidence interval (CI), 0.049–0.087).

**Figure 2:**
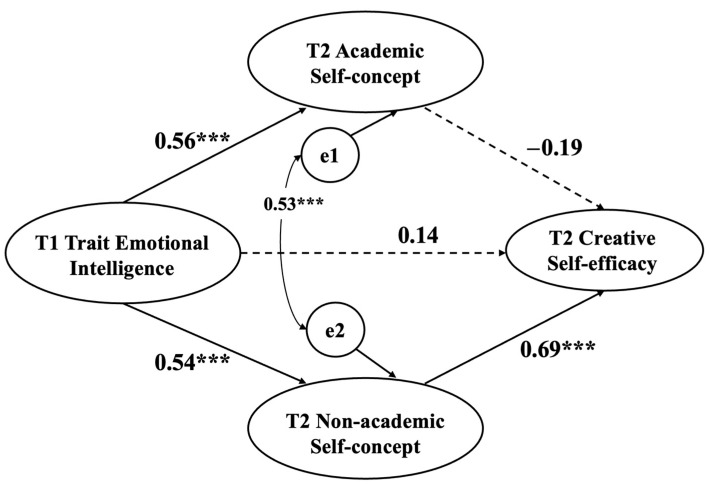
Mediating effect of T2 self-concept on the relationship between T1 trait emotional intelligence and T2 creative self-efficacy. *** *p* < .001. T1 = time 1, T2 = time 2.

The results indicated that T1 trait emotional intelligence significantly predicts T2 creative self-efficacy (β = 0.14, *p* = .13), T2 academic self-concept (β = 0.56, *p* < .001), and non-academic self-concept (β = 0.54, *p* < .001). T2 non-academic self-concept significantly predicts T2 creative self-efficacy (β = 0.69, *p* < .001), while T2 academic self-concept does not (β = −0.19, *p* = .13) (see Figure 2). The results of the mediation analysis showed that T2 non-academic self-concept has a significant mediating effect on the relationship between T1 trait emotional intelligence and T2 creative self-efficacy (95% CI [0.21~0.65], does not include 0), with an effect size of 0.38. However, T2 academic self-concept did not have a significant mediating effect on the relationship between T1 trait emotional intelligence and T2 creative self-efficacy (95% CI [−0.27~0.03], includes 0), with an effect size of −0.10 (as shown in Table 2). This result suggested that T2 non-academic self-concept fully mediates the relationship between T1 trait emotional intelligence and T2 creative self-efficacy.

## 4. Discussion

Building upon the existing research, the present study further explored the causal relationship between trait emotional intelligence and creative self-efficacy in gifted children, and we investigated the cross-temporal mediating effect of self-concept. The findings indicated that T1 trait emotional intelligence significantly and positively predicts T2 creative self-efficacy. Furthermore, T2 non-academic self-concept played a fully mediating role between T1 trait emotional intelligence and T2 creative self-efficacy.

### 4.1. Temporal Dynamics of Trait Emotional Intelligence and Creative Self-Efficacy

The findings of this study indicated that the trait emotional intelligence of intellectually gifted children at T2 was significantly lower than that at T1, while the creative self-efficacy at T2 was markedly lower than that at T1. These results support Hypothesis 1 and the BFLPE (Marsh and Parker 1984). The students who participated in this study were gifted children who were placed in experimental classes for gifted education following scientific selection. When these students compare themselves with peers who possess greater intellectual abilities, their self-concept decreases. This decline in individual self-concept is closely related to their self-assessment (Li and Shi 2005). The present study focusing on gifted children indicates that as the duration of their enrolment in gifted education programs increases, their self-concept declines (Craven et al. 2000; Duan et al. 2022; Marsh et al. 1995). When children transition from a general education class to an experimental class designed for gifted children, they quickly realize that they are no longer the center of attention within the larger group. Consequently, this realization contributes to a decline in self-concept. Moreover, there is a close association between the creative self-efficacy and trait emotional intelligence of gifted children and their self-concept. Creative self-efficacy, which is the belief in one’s ability to produce creative outcomes, is not just a reflection of one’s self-image but is intimately connected to how one evaluates their own abilities (Chen and Cheng 2023). In addition, trait emotional intelligence influences the development of an individual’s self-concept (Karwowski and Lebuda 2013). Therefore, the BFLPE may be one of the contributing factors to the diminished trait emotional intelligence and creative self-efficacy observed in gifted children. For gifted students in experimental classes, it is crucial for teachers to actively address the issues and challenges they encounter in both their academic pursuits and other aspects of life. Teachers should guide gifted children in adjusting their mindset, accurately appraising their academic achievements, handling peer pressure in a scientific manner, facing frustrations, and fostering a habit of making positive attributions.

### 4.2. Predictive Role of Trait Emotional Intelligence in Creative Self-Efficacy

The study results indicate that trait emotional intelligence at T1 significantly and positively predicted creative self-efficacy at T2, while creative self-efficacy at T1 did not predict trait emotional intelligence at T2. This suggests that there is not a mutual predictive relationship between the trait emotional intelligence and creative self-efficacy of gifted children. Trait emotional intelligence has a delayed effect on enhancing creative self-efficacy, partially supporting Hypothesis 2. The results align with social cognitive theory and the mixed model of emotional intelligence, suggesting that proficient emotional regulation skills can facilitate problem-solving, reasoning, and decision-making processes, thereby fostering the development of creative self-efficacy (He and Wong 2022). This finding is consistent with the previous research indicating that emotional experiences have a lasting impact on individual functioning (Culot and Gevers 2021; He and Wong 2022). A possible explanation is that high levels of trait emotional intelligence act as a self-regulatory resource (Bar-On 2006; Chen and Cheng 2023; Parmentier et al. 2019), enabling individuals to generate creative responses to diverse environments and tasks through the management, monitoring, and regulation of their emotions (Supervía and Robres 2021). Research also points out that trait emotional intelligence has been shown to consistently predict positive social and academic outcomes for individuals (Chen 2024). In interpersonal and social interactions, heightened trait emotional intelligence enhances individuals’ reception of support, affirmation, and recognition from the external environment, consequently reinforcing their creative self-efficacy (Puozzo and Audrin 2021).

In contrast to previous research, this study focused on a unique group of gifted children while investigating the relationship between trait emotional intelligence and creative self-efficacy. The findings highlight the enduring positive effect of trait emotional intelligence on creative self-efficacy in gifted children. As an internal psychological factor, trait emotional intelligence has significant implications for fostering the growth and success of gifted children. Gifted children possess greater cognitive abilities and creative potential, but their social development is consistent with that of their peers due to physiological age constraints (Liu and Chen 2021). In the early stages of development, enhancing gifted children’s emotional regulation, management, and expression capabilities may offset inherent developmental imbalances. Utilizing their advanced cognitive abilities and gradually reinforcing the social skills of gifted children, as well as their motivation and persistence in tasks (Barbey et al. 2014), can enable them to fully unleash their creative potential. This, in turn, leads to the creation of novel and meaningful products to enhance team performance (Chen and Shi 2013) and foster the later development of creative self-efficacy in gifted children. Therefore, it is essential for teachers and parents to nurture the trait emotional intelligence of gifted children. This involves guiding them to understand, reason, and regulate their emotions through diverse methods, aiming to enhance their positive emotional experiences and perceptions (Bar-On 2006). Simultaneously, providing more emotional support and affirmation in their daily interactions can contribute to increasing the sense of self-worth of gifted children.

### 4.3. Cross-Temporal Mediating Effect of Self-Concept

The research findings indicated that non-academic self-concept at T2 plays a fully mediating role between trait emotional intelligence at T1 and creative self-efficacy at T2. However, the cross-temporal mediating effect of academic self-concept was not statistically significant. In other words, trait emotional intelligence can facilitate creative self-efficacy through the mediation of non-academic self-concept, partially supporting Hypothesis 3. A possible explanation is that during the formation of non-academic self-concept, students with high trait emotional intelligence rely on superior emotional awareness, emotional management, and emotional regulation abilities. They keenly perceive support from others, identify positive atmospheres, transform negative emotions, and, in the process of forming a positive non-academic self-concept, creatively address challenging issues. This process fosters the development of creative self-efficacy. Furthermore, existing research has also shown that gifted children who enter experimental classes have a significant decrease in their academic self-concept, while non-academic self-concept does not change significantly (Chessor and Whitton 2005). In this study, the gifted children’s self-concept levels were measured nine months after they entered the experimental class. Influenced by the BFLPE, the decrease in their academic self-concept levels (Luo et al. 2008), may have led to a nonsignificant mediating role of academic self-concept between trait emotional intelligence and creative self-efficacy.

First, trait emotional intelligence at T1 significantly and positively promoted academic self-concept and non-academic self-concept at T2. This finding supports the previous research suggesting that the ability to handle negative emotions and maintain a positive emotional state in challenging situations helps individuals sustain a strong self-concept (Martínez-Monteagudo et al. 2021). Additionally, this finding supports social cognitive theory, as higher trait emotional intelligence signifies superior emotional management and regulation skills, contributing to enhanced self-awareness and facilitating the development of self-concept (Fernández-Berrocal and Extremera 2006). A reasonable explanation is that self-concept primarily originates from interpersonal interactions and social comparisons (Markus and Wurf 1987). Individuals derive a sense of self through their interactions with the world, thereby constructing self-concept (Duan et al. 2022). Higher trait emotional intelligence enables gifted children to effectively utilize their emotional awareness, management, and regulation abilities in interpersonal interactions. This ability helps gifted children understand the emotional cues of others, perceive support and positive emotions sensitively, express their own emotions when necessary, and contribute to the creation of a harmonious social atmosphere (Metaj-Macula 2017). This enables gifted children to receive more support and recognition from the outside world, exerting a positive impact on shaping the gifted child’s self-concept.

Furthermore, in the present study, non-academic self-concept exhibited a notable positive influence on creative self-efficacy, whereas the effect of academic self-concept on creative self-efficacy was not statistically significant. A possible explanation is that non-academic self-concepts, which include peer relationships, parent–child relationships, physical appearance, and sports, provide an important psychological foundation for creative self-efficacy by enhancing gifted children’s social support, self-esteem, and self-confidence, as well as psychological qualities such as resilience and perseverance (Bandura 1986; Cheng et al. 2023; Halford et al. 2018; Simon et al. 2022). Positive non-academic self-concepts often enable gifted children to gain more social support and recognition during their formation process. This is crucial in creative activities, aiding gifted children to collaborate with others, share ideas, and draw inspiration from feedback, further enhancing self-efficacy. In contrast, academic self-concept is mainly shaped by classroom performance, examination scores, and rankings and is often rooted in social comparisons. Traditional educational assessments commonly employ well-structured problems with clear initial states, known target states, and constrained logical factors (David et al. 2003). These problems are typically abstract and lack real-world context. Gifted students are often required to rely on a search-for-solution approach within existing frameworks (Huang 2023). As a result, gifted students have fewer opportunities for creative problem solving. This limitation diminishes the impact of academic self-concept on creative self-efficacy. It is beneficial for teachers and parents to encourage gifted children to tackle more open-ended and flexible problems. In addition, they should acknowledge and affirm the multiple creative problem-solving solutions proposed by gifted children and consider this method to be one of the criteria for evaluating their academic achievements. This approach aims to foster the positive development of gifted children’s academic self-concept while simultaneously empowering them to unleash their creative potential to the fullest.

## 5. Conclusions and Limitations

This research has significant implications for understanding the intricate dynamics between trait emotional intelligence and creative self-efficacy in gifted children, illuminating potential mechanisms involving self-concept and providing valuable insights to boost creative self-efficacy levels.

This study has some limitations that need further refinement in future research. First, the data were collected at only two time points. Future research could benefit from collecting data at multiple time points to obtain more precise and scientifically robust research outcomes. Second, this study used self-reporting to collect data, which may run the risk of subjectivity bias, which may result in less objective data. Future studies could attempt to combine subjective reports with objective tests to obtain more comprehensive and reliable results. Further, the Creative Self-Efficacy Scale only covers three questions, which may increase the risk of error variance. Future studies could choose other appropriate scales to avoid this problem. Third, the present study explored the relationships between trait emotional intelligence, self-concept, and creative self-efficacy in gifted children through a questionnaire survey. Future research could consider further clarifying the contribution of trait emotional intelligence and self-concept to creative self-efficacy using an experimental intervention design.

## Figures and Tables

**Table 1:** Mean (*M*), standard deviation (*SD*), and correlations of the variables (*n* = 177).

Variables	*M*	*SD*	1	2	3	4	5	6
1. T1_CSE	3.88	0.80	1					
2. T2_CSE	3.59	0.94	0.52 ***	1				
3. T1_TEI	3.86	0.57	0.43 ***	0.41 ***	1			
4. T2_TEI	3.76	0.58	0.31 ***	0.50 ***	0.68 ***	1		
5. T2_ASC	3.33	0.63	0.29 ***	0.28 ***	0.48 ***	0.51 ***	1	
6. T2_NASC	3.41	0.66	0.41 ***	0.51 ***	0.59 ***	0.70 ***	0.60 ***	1

Note: *** *p* < .001. T1 = time 1, T2 = time 2, CSE = creative self-efficacy, TEI = trait emotional intelligence, ASC = academic self-concept, NASC = non-academic self-concept.

**Table 2:** Results of the mediation effect test of self-concept (*n* = 177).

Effect	Predicted Path	Bootstrap 5000 95% CI		
		Effect Value	Lower	Upper
Total Effect	T1TEI→T2CSE	0.42	0.25	0.57
Indirect Effect_1	T1TEI→T2ASC→T2CSE	−0.10	−0.27	0.03
Indirect Effect_2	T1TEI→T2NASC→T2CSE	0.38	0.21	0.65
Direct Effect	T1TEI→T2CSE	0.14	−0.04	0.35

Note: T1TEI = trait emotional intelligence at time 1, T2CSE = T2 creative self-efficacy at time 2, T2ASC = academic self-concept at time 2, T2NASC = non-academic self-concept at time 2.

## Data Availability

The data are currently not publicly available due to participant privacy, but they are available from the corresponding author upon reasonable request.
